# Area normalization of HERFD-XANES spectra

**DOI:** 10.1107/S1600577524005307

**Published:** 2024-08-06

**Authors:** Luca Bugarin, Hugo Alexander Suarez Orduz, Pieter Glatzel

**Affiliations:** a ESRF – The European Synchrotron, 71 Avenue des Martyers, 38000Grenoble, France; b Ecole Doctorale de Physique Grenoble Alpes University 38400Saint-Martin-d’Hères France; c Institute for Chemical Technology and Polymer Chemistry (ITCP) Karlsruhe Institute of Technology Engesserstrasse18/20 76131Karlsruhe Germany; University of Turin, Italy

**Keywords:** HERFD-XANES, chemical sensitivity, Thomas-Reiche-Kuhn sum rule, *L*_3_-edge

## Abstract

The normalization of X-ray absorption near-edge structure (XANES) spectra is required for comparing spectral features and extracting quantitative information in analytical techniques such as linear combination analysis, principal component analysis and multivariate curve resolution. Most published data are normalized to the edge-jump, but normalization to the spectral area has also been applied. The latter is particularly attractive if only a small energy range around the absorption can be recorded reliably. Here, the two normalization methods are compared at the *L*_3_-edge of Pt, Pd and Rh, and at the Ni *K*-edge using experimental and calculated spectra. Normalization to the spectral area is found to be a viable approach if the range for the area normalization is sufficiently large.

## Abstract 2

Normalization of XANES data to the spectral area is shown to be a viable normalization method with an error of a few percent as evaluated by comparison with calculated spectra and spectra normalized to the edge-jump.

## 1. Introduction

The normalization of a XANES (X-ray absorption near-edge structure) spectrum allows comparison of spectral features and extraction of quantitative information on the system under study. Typical analysis procedures that require a well normalized dataset are linear combination analysis, principal component analysis and multivariate curve resolution (Martini & Borfecchia, 2020 ▸). Two normalization methods can be derived from quantum mechanical considerations. The edge-jump defined as the cross section for photoionization, *i.e.* excitation into the continuum, from a given energy level (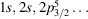) in an atom is independent of the chemical environment. Normalization of spectra to the edge-jump therefore allows comparison between spectra of different compounds. The recommended procedure to acquire a XAFS (X-ray absorption fine structure) spectrum is thus to collect the spectrum over a sufficiently long energy range, usually at least 150–200 eV below the edge energy (*e*_0_) and 400 eV above, for an optimal pre- and post-edge fitting followed by a robust edge-jump normalization (Calvin, 2013 ▸). This good practice, however, is not always applicable because of experimental constraints. For instance, in the field of catalysis sometimes the acquisition range may be limited to a few tens of eV above *e*_0_ to allow monitoring a reaction with a time resolution below its reaction rate and at the same time still obtain a good quality XANES spectrum with a high signal-to-noise (S/N) ratio. Shortening the XANES collection energy range may also be necessary when the absorber concentration is very low or the absorber is embedded in a high-*Z* matrix. The presence of unwanted absorption edges may limit the range for pre- or post-edge fitting. The short absorption length in the tender X-ray range and the large angular range that has to be scanned by crystal monochromators often limits the achievable spectral quality and pre- and post-edge can only be fitted with a large uncertainty (Lamberti & Van Bokhoven, 2016 ▸). Alternative to edge-jump normalization, spectra can be normalized to the integrated differential oscillator strength (Olney *et al.*, 1997 ▸). This method is based on the Thomas–Reiche–Kuhn (TRK) (or *f*-) sum rule that states that the sum over all dipole transition matrix elements divided by the transition energy is proportional to the total number of electrons in the system (Thomas, 1925 ▸; Reiche & Thomas, 1925 ▸; Savasta *et al.*, 2020 ▸). Its applicability to one absorption edge would require validation of the TRK sum rule for one energy level which is beyond the mathematical scope of the present authors. Therefore, we chose to compare spectral area normalization with spectra normalized to their edge-jump and with calculated spectra. Spectra recorded in high-energy-resolution fluorescence detection (HERFD) mode in most cases have negligible background such that pre-edge fitting is not required. This greatly facilitates normalization to the spectral area rendering this method a very attractive tool.

## 2. Normalization methods

### 2.1. Edge-jump normalization method

Normalization to the edge-jump requires the XAS spectrum to be collected over a sufficiently long energy range both below and above *e*_0_. The steps of this normalization method have been reported in many publications (Calvin, 2013 ▸; Newville, 2013 ▸, 2014 ▸; Ravel & Newville, 2005 ▸). First, *e*_0_ is determined usually as the maximum of the first derivative of the spectrum at the lowest energy neglecting possible pre-edge spectral features. Low-order polynomial lines are fitted in the energy region below and above *e*_0_ to the spectra to describe the pre-edge and post-edge regions. The edge-jump [Δμ_0_(*e*_0_)] is determined by taking the difference of the extrapolated values of the pre-edge and post-edge lines at the *e*_0_ value. The spectrum is finally normalized by subtracting the pre-edge line over the whole spectra range and dividing the data by Δμ_0_(*e*_0_) (Ravel & Newville, 2005 ▸; Newville, 2013 ▸). Weng *et al.* proposed a normalization method (*MBACK*) that is more robust when only the XANES region is available in the experimental data (Weng *et al.*, 2005 ▸). In this case, normalization to the edge-jump may introduce unphysical curvature in the edge or post-edge regions. Briefly, the *MBACK* method relies on a minimization algorithm that calculates a smooth background function and matches the experimental data to the tabulated values of the imaginary part of the energy-dependent correction to the Thompson scattering factor [*f*′′(*E*)] (Weng *et al.*, 2005 ▸; Lee *et al.*, 2009 ▸). This method is mathematically more robust than the edge-jump normalization but it is not adapted for the normalization of XANES data collected in HERFD detection mode. HERFD-XANES data generally have a weak, flat pre-edge signal. Thus, if the background function is subtracted from the HERFD-XANES data it will introduce a positive or negative slope to the pre-edge region, ultimately leading to an incorrect normalization of the data. Moreover, some normalization programs use a core-hole broadened corrected *f*′′(*E*) to have a better match of the standard XANES spectrum (transmission or fluorescence) (Newville, 2013 ▸). In the case of HERFD-XAS data, this correction would need to be adapted case-by-case because of the reduced lifetime broadening and consequently increased contribution of instrumental broadening (Glatzel & Bergmann, 2005 ▸; Hämäläinen *et al.*, 1991 ▸). We note, however, that it is in principle possible to modify the *MBACK* algorithm to satisfy the specifics of a HERFD-XANES spectrum, *i.e.* fit only the post-edge region to the calculated cross section.

### 2.2. Step function normalization and curve fitting approaches

HERFD-XANES data do not require a pre-edge background subtraction. Therefore, a basic approach to normalize HERFD-XANES data could be to fit the XANES spectrum with a step function (*e.g.* arctangent or simple error function) that represents the excitations into the continuum. In this case, the step height will represent the Δμ_0_ to which each spectrum has to be normalized. This approach is only valid if the fit of the step function is performed in a limited XANES data range where it can be assumed that the decrease of the continuum absorption cross-section can be neglected. This could be improved by an adapted *MBACK* algorithm.

The positioning of the onset of the step function has to be selected case by case since no general rule to identify the edge energy can be established. Indeed, the result of the fitting procedure will strongly depend on the spectral shape in the white line region. The adverse effect of the white line features in the determination of the edge-jump could be mitigated by fitting the white line features with a set of Gaussians, Lorentzian or pseudo-Voigt functions (Petit *et al.*, 2001 ▸; Henderson *et al.*, 2014 ▸). However, identifying an identical set of fitting parameters for a large set of spectra is challenging and likely will introduce a significant error.

### 2.3. Area normalization

A different approach for the normalization of HERFD-XANES data is to normalize the spectra to their area in a given energy range. This method is commonly used to compare core-to-core X-ray emission spectra where it is reasonable to assume that the sum over all dipole transition probabilities between two core hole configurations (*e.g.* 1*s*^1^3*p*^6^ to 1*s*^2^3*p*^5^ for *K*β lines) is independent of the oxidation state and chemical environment (Lafuerza *et al.*, 2020 ▸; Henthorn & DeBeer, 2022 ▸; Mathe *et al.*, 2019 ▸). Extending this approach to the analysis of HERFD-XANES is tempting but theoretically not well established to our knowledge. The TRK sum rule states that the total integrated oscillator strength for a bound electron transition in an atom or molecule (*i.e.* the sum of the squares of the transition dipole matrix elements) is constant if the number of electrons in the system remains unchanged. X-ray absorption probes unoccupied orbitals or electron density of states that are strongly modified by the chemical environment.^1^ Oxidation or reduction formally changes the charge on the absorber atom. However, XANES probes orbitals up to tens of eV above the absorption edge, thus probing orbitals that are delocalized over several atoms. A sum rule would mean that dipole transition probability is not lost or added upon oxidation, reduction or any change of ligand environment but rather redistributed. This redistribution of electron density is captured by recording XANES data over a sufficiently large energy range. We do not know if this is the case and we therefore test this hypothesis by comparing the area with edge-jump normalization in the following.

The normalization is performed simply by dividing a raw spectrum *S*_raw_ by the integrated area in a selected energy region,
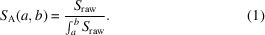
The normalization energy range has to be chosen. We assume that a correct normalization of a series of XANES spectra is possible when equation (1) is applied over a sufficiently wide energy range such that all redistributed oscillator strength is included. The area of the XANES spectrum thus represents the total integrated oscillator strength for a given core-hole energy level of the target element scaled to the concentration of absorbing atoms.

## 3. Comparison between normalization approaches

Estimating the variation between different normalization methods requires the definition of a figure-of-merit. In the case of comparison between edge-jump normalization methods (*e.g.* edge-jump normalization, *MBACK* and step function normalization), the variation can be estimated simply by comparing the magnitude of the Δμ_0_ retrieved with each method. However, when comparing HERFD-XANES spectra that are edge-jump-normalized to area-normalized ones, it is necessary to define a common metric that can describe the variation depending on the chosen normalization range, which may be arbitrarily selected.

### 3.1. Methodology to compare normalization approaches

The objective of any normalization is to be able to compare spectra. We therefore have to define a figure-of-merit that quantifies the spectral difference between two spectra *S*^1^ and *S*^2^ both normalized by the same method. We introduce a normalized area difference parameter (NAD) defined as follows,
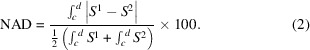
*S*^1^ and *S*^2^ are two spectra of a set of spectra where all spectra are normalized by the same method. The parameters *c* and *d* are the start and end energy points over which an integration is performed to calculate the NAD. This energy range defines what spectral features are included in the comparison. In this way, we obtain values for NAD for pairs of spectra *S*^1^ and *S*^2^  from a given set of data. For our purposes, one set of spectra is normalized to the area (A) and another set is normalized either to the edge-jump or, in case we compare calculated spectra, maintains the calculated oscillator strength without any normalization. We refer to the latter set as the standard (S) normalized spectra. We then calculated the percentage variation between two normalization approaches as
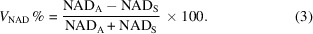
This percentage allows us to assess the error that the different normalization methods introduce. Using this definition, we were able to calculate the 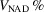 values as a function of the area normalization range selected for the (A) dataset. This was done by computing values for NAD_A_ for sets of spectra that were area-normalized using different energy ranges, *i.e.* different values for *b* in equation (1). Inserting equation (1) into equation (2) gives
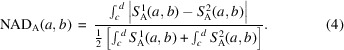
The value for NAD thus becomes a function of the parameters that were chosen for the area normalization. In our analysis, we always set parameter *a* to be fixed to the first incident energy point of the raw spectrum while *b* was varied between 20 and 100 eV above the value for *e*_0_ of one of the two raw spectra. The NAD integration ranges *c* and *d* were instead always kept fixed to −10 eV and 40 eV, respectively, relative to the *e*_0_ value. This implies that the area normalization range would end at times before (*b* < *d*) and after (*b* > *d*) the NAD_A_ integration ranges.

## 4. HERFD-XANES measurement details

The Pt *L*_3_-edge HERFD-XANES spectra were collected at ESRF beamline ID26. PtO_2_ and PtO_2_·6H_2_O were measured in different beam times and in both experiments reference Pt foil spectra were acquired. For both experiments the third harmonic of three undulators (u35) produced the incoming radiation, which was monochromated by a pair of cryogenically cooled Si(111) crystals. The X-rays were focused by two bent Pd-coated Si mirrors (at 2.5 mrad) to a size of ∼600 µm × 100 µm (horizontal × vertical) at the sample position. The ID26 hard X-ray emission spectrometer was employed (Glatzel *et al.*, 2021 ▸). For PtO_2_, three spherically bent (*R* = 1 m) Ge (660) analyser crystals (Rowland geometry) were utilized to select the fluorescence at the maximum of the Pt-*L*α_1_ emission line (9442.9 eV). Five spherically bent (*R* = 1 m) Ge (660) analyser crystals were used instead for the measurement of PtO_2_·6H_2_O, selecting the Pt-*L*α_1_ emission line (9442.79 eV). In both cases, the data were recorded with an avalanche photodiode detector, while the incident energy was scanned around the Pt-*L*_3_ absorption edge (11564 eV).

## 5. Comparison with edge-jump normalization

**Figure 1:**
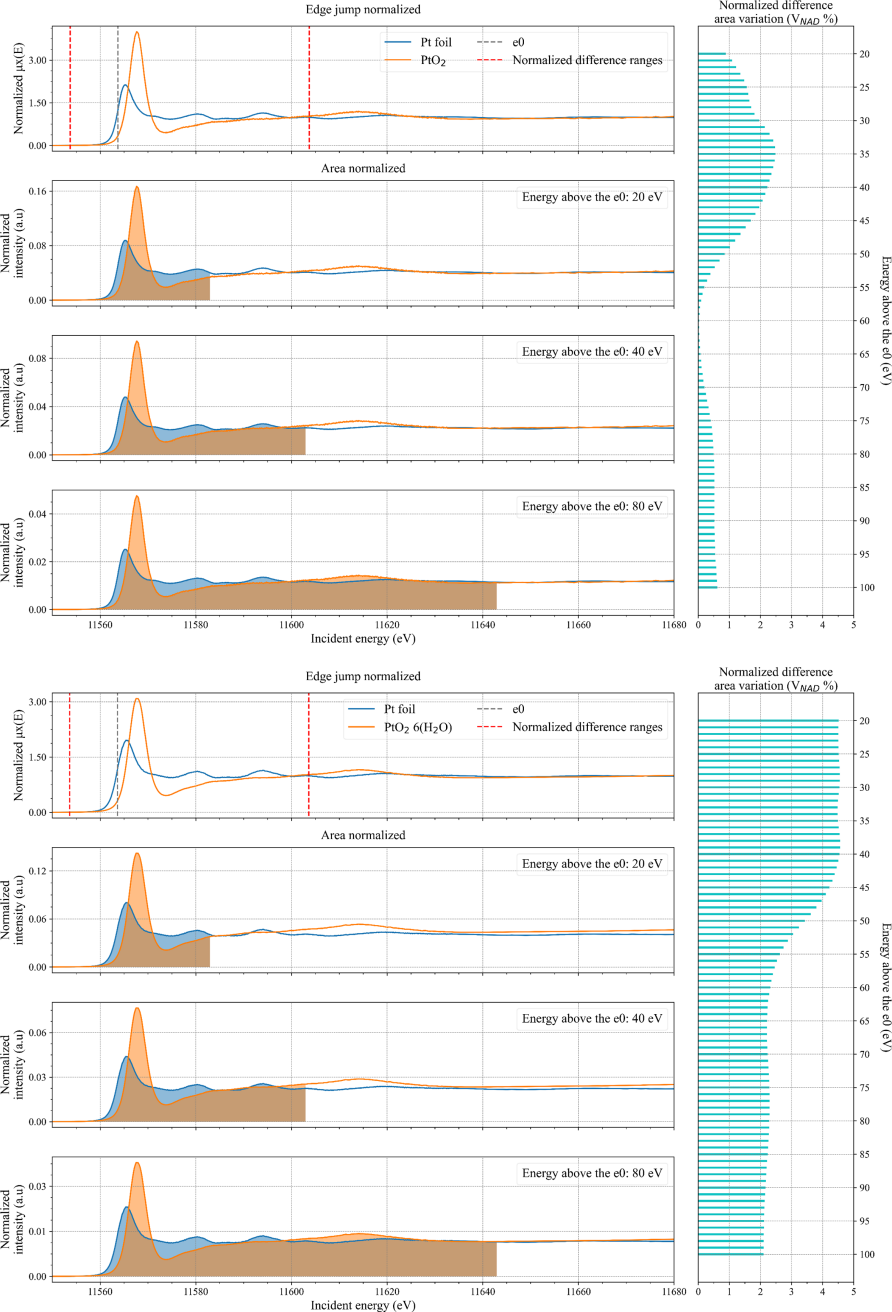
(Left) Comparison between edge-jump and area normalization for experimental data of Pt foil (blue) and, respectively, PtO_2_ (top panels) and PtO_2_·6H_2_O (bottom panels) (orange). The grey dotted line represents the position of *e*_0_ of the Pt foil spectrum that is used to determine the integration ranges (red dotted lines) over which 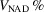 was calculated. The spectra for area normalization up to 20, 40 and 80 eV above *e*_0_ are reported and the respective areas have been coloured. (Right) Horizontal bar plot of the calculated 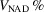 values for edge-jump versus area normalization as a function of integration end energy [parameter *b* in equation (1)].

We compare Pt HERFD-XANES data normalized to the spectral area with the conventional edge-jump normalized data and express the variation in terms of 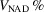. The results are shown in Fig. 1 ▸. For the comparison, we compared the Pt *L*_3_-edge HERFD-XANES spectra of a Pt reference foil with those of PtO_2_ and PtO_2_·6H_2_O pellet samples. PtO_2_ and PtO_2_·6H_2_O references were selected to investigate the case in which, despite a Pt nominal oxidation number of 2+, spectra may differ in number of unoccupied states, as can be seen by the variation in white line intensity between the two spectra (Fig. S1 of the supporting information). All the XANES spectra were collected in the energy range between 11500 and 12100 eV. All the spectra were first interpolated on a fine grid with a step size of 0.1 eV. The normalization to the edge-jump was performed using the Larch Python library version_0.9.74 over the whole data range (Newville, 2013 ▸). The *e*_0_ value was set to be equal to the first maximum of the first derivative of the spectrum. For all the spectra, both pre-edge and post-edge regions were fitted with a first-order polynomial function in the energy ranges from −65 to −35 eV and from 90 to 475 eV with respect to *e*_0_. The NAD_S_ for these spectra was calculated using the normalized signal without flattening the spectra.

Area normalization of the spectra was performed letting the maximum energy value for the area integration range from 20 up to 100 eV above *e*_0_ of the Pt foil (11563.62 eV) with a step of 1 eV. For each couple of normalized spectra in a given area range, we calculated NAD_A_. The 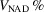 value was calculated between −10 and 40 eV (parameters *c* and *d*) relative to the *e*_0_ value of the Pt foil.

**Figure 2:**
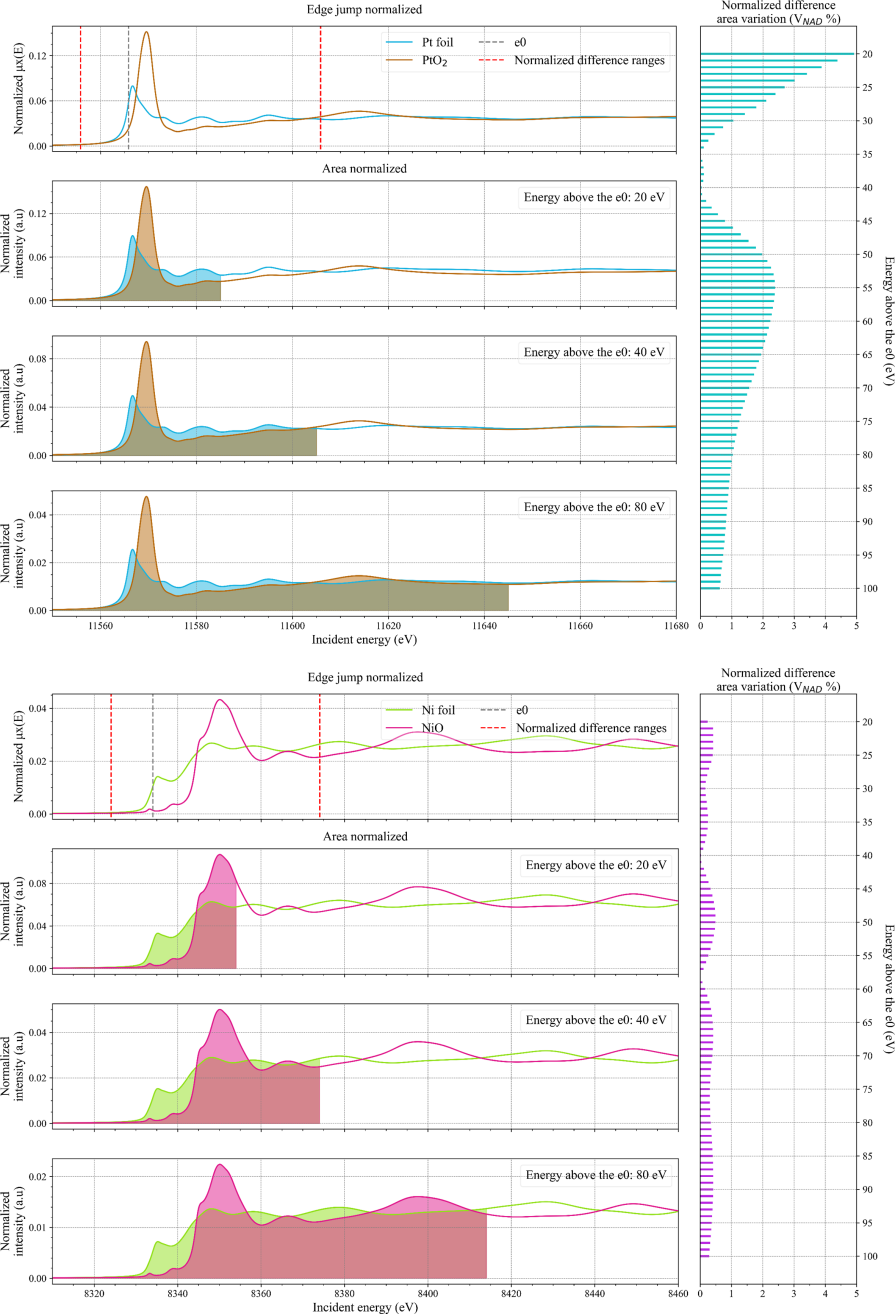
(Left) Comparison between *FDMNES* simulated spectra and the same spectra normalized by area for Pt (light blue line) and PtO_2_ (brown line) in the top panels and Ni (light green line) and NiO (magenta line) in the bottom panels. The grey dotted line represents the position of *e*_0_ of the metallic Pt and Ni spectrum used to determine the integration ranges (red dotted lines) over which 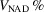 was calculated. The plots for area normalization of 20, 40 and 80 eV above *e*_0_ are reported and the respective areas are coloured. (Right) Horizontal bar plot of the calculated 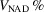 values for edge-jump versus area normalization as a function of integration range.

The analysis shows that the XANES spectra can be well normalized by area yielding results that are quite similar to those obtained with the edge-jump normalization method. Normalizing the XANES spectra by area using an integration range after *e*_0_ between 20 and 100 eV results in only a small variation of less than 5% in all the considered ranges (Figs. 2 ▸ and 3 ▸). As expected, the higher the value above *e*_0_ for the area normalization, the lower 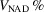 as the contribution from continuum excitations increases. We note that by using the same figure-of-merits one could compare different edge-jump normalizations, *e.g.* using different orders for the post-edge polynomial function and fitting ranges. This is beyond the scope of the current study, but we assume that the error is similar to what we obtain here for area normalization.

## 6. Comparison with *FDMNES* simulation

### 6.1. *L*_3_-edges

We propose a further comparison to test the validity of the area normalization method using calculated XANES spectra (Nascimento & Govind, 2022 ▸). Comparing the simulated XANES spectra that arise from the calculated oscillator strength and normalizing the same spectra by area will tell us about the amount of variation that is introduced by using the area normalization approach. The simulated XANES spectra were obtained using the *FDMNES* code version_2023.04.27 (Joly *et al.*, 2009 ▸). *L*_3_-edge simulations were obtained for metallic Pt, PtO_2_ Pd, PdO, metallic Rh and RhO_2_ references. The details of the parameters of the calculations and of the convolution parameters are reported in Section S2 of the supporting information. The simulated spectra were blue-shifted to match the experimental energy and interpolated over a finer energy grid of 0.1 eV (Section S3). The same methodology used for the comparison with the edge-jump method was applied. The maximum energy value for the area integration range was selected to range between 20 up to 100 eV above *e*_0_ of the metallic Pt (11565.85 eV), metallic Pd (3175.20 eV) or metallic Rh (3005.15 eV) simulated spectra. The incremental step for each calculation of the NAD_A_ was 1 eV. Finally, 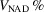 was calculated for all simulations selecting as normalization difference ranges −10 and 40 eV below and above *e*_0_ of each metallic spectrum (parameters *c* and *d*).

**Figure 3:**
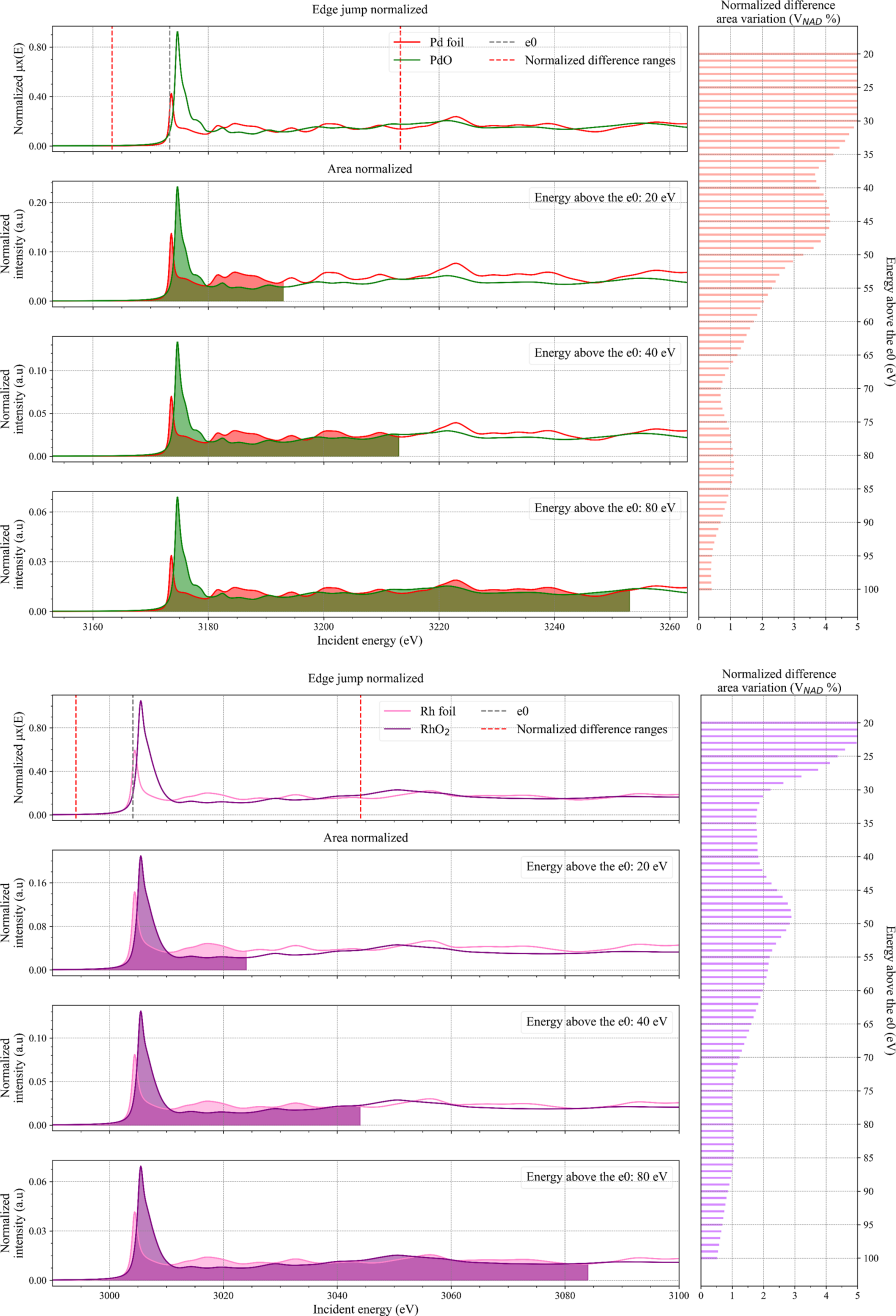
(Left) Comparison between *FDMNES* simulated spectra and the same spectra normalized by area for Pd (green line) and PdO (red line) in the top panels and Rh (pink line) and RhO_2_ (purple line) in the bottom panels. The grey dotted line represents the position of *e*_0_ of the metallic Pd and Rh spectrum used to determine the integration ranges (red dotted lines) over which 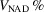 was calculated. The plots for area normalization of 20, 40 and 80 eV above *e*_0_ are reported and the respective areas have been coloured. (Right) Horizontal bar plot of the calculated 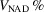 values for edge-jump versus area normalization as a function of integration range.

The results obtained for the Pt simulations (Fig. 2 ▸, top) show that 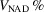 is below 10% in the whole area integration range (20 to 100 eV). The value for 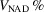 strongly varies for small integration ranges mainly because of the high amplitude of the oscillation immediately after the white line region (*e.g.* broad feature at around 11612 eV in the PtO_2_ spectrum; Fig. 2 ▸, top). The 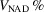 value steadily decreases with the attenuation of the EXAFS oscillation at higher energies. The same behaviour is observed for the Pd and Rh simulations that are reported in Fig. 3 ▸. The values for 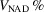 are on average higher with respect to the Pt simulations, but always below 5% for integration ranges above 35–40 eV with respect to *e*_0_, *i.e.* the region where the EXAFS oscillations start to become weaker.

### 6.2. 3*d* transition metal *K*-edges

We studied area normalization also for *K*-edges of 3*d* transition metals (TMs). For *K*-edges of 3*d* transition metals, oxidation and reduction should have little effect on the integrated oscillator strength because the 3*d* orbitals can only be probed via quadrupole transitions that give rise to the weak pre-edge features. Therefore, area normalization of a XANES spectrum is expected to be a viable approach if a sufficiently large normalization range is considered. We used Ni and NiO as reference compounds for this study. *FDMNES* simulations for the two compounds were interpolated on a finer grid of 0.1 eV and aligned to match the energy of the experimental spectra. The Ni simulations were analysed with the same parameters used for the *L*_3_-edge simulations with *e*_0_ of the metallic Ni being at 8333.37 eV. The 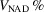 analysis shows that the amount of error introduced via area normalization is well below 1% for all the considered integration ranges (Fig. 2 ▸, bottom). This smaller error already for short normalization ranges compared with the *L*_3_-edge analysis may be explained by the smaller contribution of the valence orbitals to the integrated spectral intensity as discussed above.

Close inspection of the spectra shows that in the region between 8360 and 8380 eV the agreement improves for longer normalization ranges. We always use the same parameters *c* and *d* to calculate 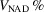 and the error may increase if a different spectral range is considered.

## 7. Conclusions

Analysis of HERFD-XANES spectra at the *L*_3_-edge of Pt, Pd and Rh compounds and the *K*-edge of Ni compounds suggests that the area normalization is a valid approach when a sufficiently large energy range is selected (at least 30 eV above *e*_0_). Normalization to the spectral area is valid for compounds with different oxidation states and when the valence orbitals are probed via dipole transitions. This may be counterintuitive as the number of valence electrons in the absorber atom formally changes upon oxidation thus invalidating the sum rule. A possible explanation would be that oxidation and reduction processes shuffle intensity between more localized and delocalized (from the absorber ion point of view) orbitals that are all probed in the X-ray absorption process leaving the integrated oscillator strength sufficiently invariant.

Based on our analysis results using area normalization, it is recommended to err on the side of caution by choosing a total range from −10 up to 80–100 eV with respect to *e*_0_. This selection will ensure to include both potential pre-edge region features and a portion before the edge for background subtraction. However, care should be taken not to extend this range excessively, since no polynomial is fitted to account for the decrease of the continuum absorption cross-section.

We note that any normalization method introduces an error in the data analysis. We think normalization to the spectral area does not significantly increase this error compared with normalization to the edge-jump which also depends on the chosen parameters. It is important to take this error into account in any multi-variate analysis including machine learning algorithms. Furthermore, HERFD-XANES data are distorted by over-absorption whose magnitude depends on the absorber concentration and the matrix. This adds to the error in spectral normalization if the distortion varies between the compared spectra.

## Supplementary Material

Sections S1 to S3, including Tables S1 to S3 and Figures S1 to S3. DOI: 10.1107/S1600577524005307/uc5004sup1.pdf

## Footnotes

1 In the following, we use the term ‘orbital’ also for electron levels in the continuum. This is motivated by the fact that the photoelectron experiences the potential of the photoionized as well as the surrounding atoms and is thus not free. The potentials give rise to the EXAFS oscillations. We furthermore note that photoionization follows selection rules just like resonant excitations. We think that describing continuum excitations as excitations into delocalized ‘orbitals’ is in the present context a useful concept (Breinig *et al.*, 1980 ▸).
