# Supplementary material for: Area normalization of HERFD-XANES spectra
Source: J Synchrotron Radiat. 2024 Aug 6;31(Pt 5):1118–25. doi: 10.1107/S1600577524005307 (PMC11371039; doi:10.1107/S1600577524005307)
Supplement: Supplementary file 1 [file s-31-01118-sup1.pdf]

JOURNAL OF SYNCHROTRON RADIATION

**Volume 31 (2024)**

**Supporting information for article:**

# Spectral area normalization of HERFD-XANES spectra

**Luca Bugarin, Hugo Alexander Suarez Orduz and Pieter Glatzel**

### S1. Comparison between edge-jump normalization approaches

To make a comparison between normalization approaches in the case of spectra collected in a shorter data range the same raw HERFD-XAS spectra were cut in the XANES energy range between 11540 and 11650 eV and normalized using the step function approach with and without fitting the white line region with a single Voigt function and compared to the previously edge-jump normalized spectra in the full energy range (11500-12300 eV). In both cases the $e^0$ was set to correspond to the first maximum of the first derivative of the XANES spectra. The step was modelled using the python lmfit library version_1.2.2 trying both using an arctangent or an error function. The latter gave overall better and consistent results during the fitting procedure of all the XANES spectra. The center of the step function was imposed equal to the $e^0$ value for each spectrum, the curvature of the step ("sigma" value) was fixed to 0.25 and only the "step_amplitude" parameter was allowed to vary. For the fitting with the Voigt function, the Lorentizan "gamma" parameter was fixed equal to 0.1, while the Gaussian "sigma" parameter was left varying, as well as the amplitude value and the center position. Finally, for both cases, the data was then divided by the "step_amplitude" value retrieved from the best fit and the $NAD_S$ was evaluated.

The full width at half maximum (FWHM) of the fitting procedure of the step together with Voigt functions is reported in Table S1. In Table S2 and S3 the $\Delta\mu_0$ obtained using the different normalization methods is reported.

**Table S1** FWHM of the Voigt functions.

| Pt foil[1] | $PtO_2$ | Pt foil[2] | $PtO_2$•6($H_2O$) |
|---|---|---|---|
| 3.97 | 3.37 | 3.34 | 3.59 |

The superscript [1] and [2] refers to two different Pt XANES spectra collected in separate beamtimes for $PtO_2$ and $PtO_2$•6($H_2O$) respectively.

**Table S2** Edge-jump values ($\Delta\mu_0$) were obtained with edge-jump, and step function normalization methods.

| | Pt foil[1] | $PtO_2$ | Pt foil[2] | $PtO_2$•6($H_2O$) |
|---|---|---|---|---|
| Edge-jump | 1.31 | 0.72 | 8.12 | 7.06 |
| Step | 1.40 | 0.78 | 8.65 | 7.36 |
| Step + Voigt | 1.33 | 0.69 | 8.23 | 6.62 |

We calculated the percentage variation on the $\Delta\mu_0$ values (Table S3) as:

$$Percentage\ variation = 1 - \frac{\Delta\mu_{0_{step\ norm}}}{\Delta\mu_{0_{edge-jump\ norm}}}$$

**Table S3** Percentage variation of the $\Delta\mu_0$ values of the step normalization approach with respect to the edge-jump normalization.

| | Pt foil[1] | $PtO_2$ | Pt foil[2] | $PtO_2$•6($H_2O$) |
|---|---|---|---|---|
| Step | -7% | -8% | -7% | -4% |
| Step + Voigt | -2% | 4% | -1% | 6% |

For all the cases, the variation is below 10%, but overall, the curve fitting approach (Step + Voigt) gives values closer to the one estimated with the edge-jump normalization approach.

DOIs:

Ni data: https://doi.esrf.fr/10.15151/ESRF-ES-879295211. Pt data: https://doi.esrf.fr/10.15151/ESRF-ES-1017380304.

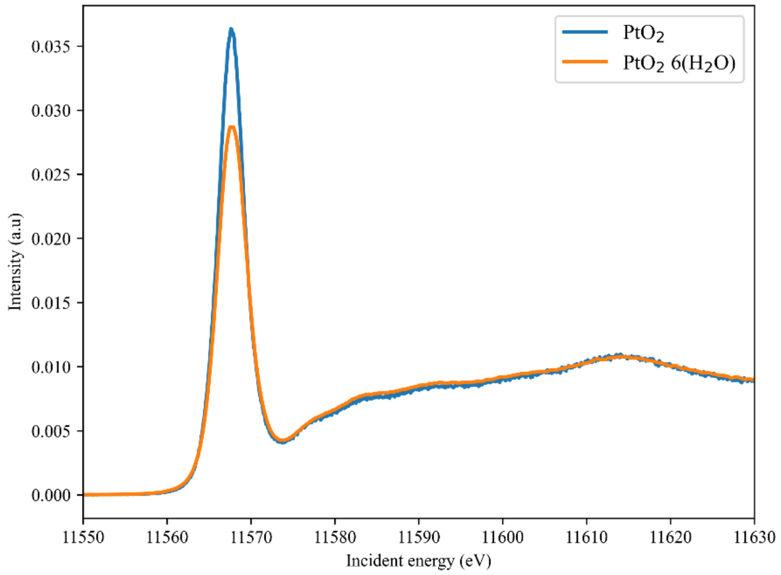


**Figure S1** Comparison of $PtO_2$ and $PtO_2$ $6H_2O$ HERFD-XANES experimental spectra.

### S2. FDMNES simulation

FDMNES input file - Pt metallic cif file ICSD 243678

```
Filout
out

Full_atom
SCF
Radius
9
Range

-30 0.2 -10 0.02 0 0.05 15 0.1 30 0.25 50 0.5 60 1 117

Edge
L3

Spinorbit
Cif_file
Pt_foil.cif

Absorber

Quadrupole

Convolution
Ecent
35
Elarge
20
Gamma_max
10
Gamma_hole
2.2
Ecut
-0.1
Estart
-30
```

FDMNES input file – $PtO_2$ cif file ICSD 1008935

```
Filout
out
Full_atom
SCF
Radius
5
Range
-10 0.02 8 0.05 15 0.5 30 1 117

Edge
L3
Spinorbit

Cif_file
PtO2_1008935.cif

Absorber

Quadrupole

Convolution

Ecent
35
Elarge
20
Gamma_max
10
Gamma_hole
2.2
Ecut
1.5
Estart
-30
```

FDMNES input file – Pd metallic

```
Filout
Pd-foil-17Apr-R9
Range
-5. 0.1  5. 0.25 20. 0.5 100.

Edge
L3

Spgroup
Fm-3m
SCF
Relativism
Spinorbit
Excited
Density
Green
Radius
9
Crystal
        3.957066 3.957066 3.957066 90 90 90
  46  0.00000000  0.00000000  0.00000000
  46  0.00000000  0.50000000  0.50000000
  46  0.50000000  0.00000000  0.50000000
  46  0.50000000  0.50000000  0.00000000

Convolution
Gamma_hole
0.6
Gamma_max
3.5
Estart
-20
```

FDMNES input file – PdO

```
Filout
PdO-17Apr-R9
Range
-5. 0.1  5. 0.25 20. 0.5 100.
Edge
L3

SCF
Spgroup
131
Relativism
Spinorbit
Excited
Density
Green

Radius
9
Crystal
3.06000 3.06000 5.37200 90 90 90
46      0.500000        0.000000        0.000000
8       0.000000        0.000000        0.250000

Convolution
E_cut
-0.79011
Ecent
30
Elarg
10
Gamma_hole
0.9
Gamma_max
3.5
Estart
-20
```

FDMNES input file – Rh metallic

```
Filout
Rhfoil-17Apr-R9
Range
-5. 0.1  5. 0.25 20. 0.5 100.
Edge
L3

Spgroup
Fm-3m
SCF
Relativism
Spinorbit
Excited
Density
Green

Radius
9
Crystal
         3.80340 3.80340 3.80340 90 90 90
  45  0.00000000 0.00000000 0.00000000

Convolution
E_cut
-0.1
Ecent
2
Elarg
10
Gamma_hole
1
Gamma_max
3.5
Estart
-20
```

FDMNES input file – $RhO_2$

```
Filout
RhO2-17Apr-R9
Range
-5. 0.1  5. 0.25 20. 0.5 100.
Edge
L3

SCF
Relativism
Spinorbit
Excited
Density

Green

Radius
9
Crystal
4.4862 4.4862 3.0884 90 90 90
45      0.000000        0.000000        0.000000
45      0.500000        0.500000        0.500000
8       0.306730        0.306730        0.000000
8       0.806730        0.193270        0.500000
8       0.193270        0.806730        0.500000
8       0.693270        0.693270        0.000000

Convolution
E_cut
-0.1
Ecent
5
Elarg
10
Gamma_hole
1
Gamma_max
3.5

Estart
-20
```

FDMNES input file – Ni metallic cif file ICSD 37502

```
Filout
out

Full_atom
SCF
Density
Radius
8
Range
-30 0.2 -10 0.02 0 0.05 15 0.1 30 0.25 117 0.5 200

Edge
K
Cif_file
Ni_37502.cif

Absorber

Quadrupole

Convolution
```

FDMNES input file – NiO cif file ICSD 9866

```
Filout
out

Screening
0.1
Full_atom
SCF
Radius
6
Range
-30 0.2 -10 0.02 0 0.05 15 0.1 30 0.25 117 0.5 200

Edge
K

Cif_file
NiO_9866.cif

Absorber

Quadrupole

Convolution
E_cut
-6
Gamma_max
15
Ecent
40
Elarge
30
```

### S3. Alignment of FDMNES simulated with experimental spectra

All FDMNES simulations were interpolated on a finer grid of 0.1eV and aligned manually by a fixed amount for each element to match the experimental spectra. To allow a direct comparison of the simulated spectra, we had to divide each calculated spectrum by its total number of symmetrical absorber atoms in the unit cell (the number can be found under the "Symatom" line in the FDMENS output). Ni foil and 0.5% NiO in cellulose pellet sample were measured at the ESRF ID26 beamline using a set of 5 spherically bent (R=1 m) Ge (620) crystal analyzers. HERFD-XANES spectra were collected at the Ni-K$\alpha_1$ at (7479.5 eV).

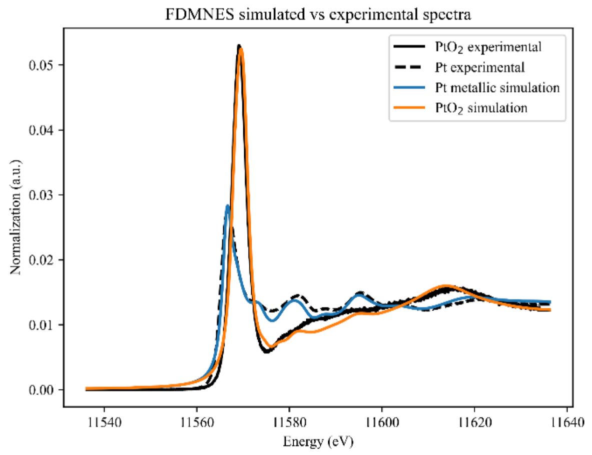


**Figure S2** Comparison between Pt and $PtO_2$ experimental spectra and FDMNES simulations.

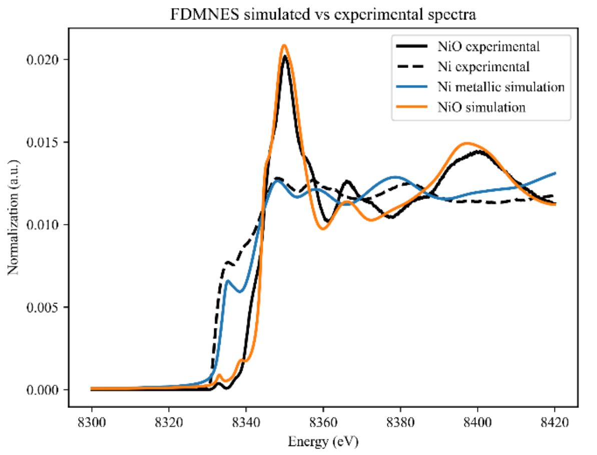


**Figure S3** Comparison between Ni and NiO experimental spectra and FDMNES simulations.between Ni and NiO experimental spectra and FDMNES simulations.
